# Role of the Lipid Membrane and Membrane Proteins in Tau Pathology

**DOI:** 10.3389/fcell.2021.653815

**Published:** 2021-04-30

**Authors:** Eugene Bok, Eunju Leem, Bo-Ram Lee, Ji Min Lee, Chang Jae Yoo, Eun Mi Lee, Jaekwang Kim

**Affiliations:** ^1^Dementia Research Group, Korea Brain Research Institute, Daegu, South Korea; ^2^School of Life Sciences, Kyungpook National University, Daegu, South Korea; ^3^Department of Brain and Cognitive Sciences, Daegu Gyeongbuk Institute of Science and Technology, Daegu, South Korea

**Keywords:** tau, tauopathy, membrane, transmission, aggregation

## Abstract

Abnormal accumulation of misfolded tau aggregates is a pathological hallmark of various tauopathies including Alzheimer’s disease (AD). Although tau is a cytosolic microtubule-associated protein enriched in neurons, it is also found in extracellular milieu, such as interstitial fluid, cerebrospinal fluid, and blood. Accumulating evidence showed that pathological tau spreads along anatomically connected areas in the brain through intercellular transmission and templated misfolding, thereby inducing neurodegeneration and cognitive dysfunction. In line with this, the spatiotemporal spreading of tau pathology is closely correlated with cognitive decline in AD patients. Although the secretion and uptake of tau involve multiple different pathways depending on tau species and cell types, a growing body of evidence suggested that tau is largely secreted in a vesicle-free forms. In this regard, the interaction of vesicle-free tau with membrane is gaining growing attention due to its importance for both of tau secretion and uptake as well as aggregation. Here, we review the recent literature on the mechanisms of the tau-membrane interaction and highlights the roles of lipids and proteins at the membrane in the tau-membrane interaction as well as tau aggregation.

## Introduction

Tau is a microtubule (MT)-associated protein that plays important roles in regulating MT dynamics and cellular trafficking as well as signaling pathways (Morris et al., 2011; Barbier et al., 2019). Abnormal accumulation of tau aggregates is a common pathological hallmark of many tauopathies including Alzheimer’s disease (AD) (Kosik et al., 1986). Although tau is a natively highly soluble unstructured protein (Jeganathan et al., 2008; Mukrasch et al., 2009), it undergoes a variety of post-translational modifications (PTMs) and conformational changes, leading to accumulation of pathological tau species, such as toxic tau oligomers and fibrils, under pathological conditions (Wang and Mandelkow, 2016). Tau pathology in AD patients shows a unique spatiotemporal propagation from entorhinal cortex to hippocampus and neocortex, which is closely correlated with cognitive decline in AD (Braak and Braak, 1991; Vogels et al., 2020). Therefore, understanding the mechanisms underlying aggregation and spreading of tau may provide new opportunities to develop novel therapeutic interventions for tauopathies.

Pathological tau aggregates as well as physiological tau monomer can be released from the donor cells and taken up by recipient cells (De La-Rocque et al., 2020). Tau secretion involves multiple different pathways, such as vesicle-mediated pathways through exosome (Lee et al., 2012), ectosome (Dujardin et al., 2014), and vesicle-free direct translocation across the plasma membrane (PM) (Katsinelos et al., 2018; Merezhko et al., 2018). Similarly, tau uptake also involves many different pathways, such as endocytosis (Frost et al., 2009), macropinocytosis (Holmes et al., 2013), and phagocytosis (Brelstaff et al., 2018). The pathways of tau secretion and uptake are reviewed in detail in recent publications (Pernegre et al., 2019; Brunello et al., 2020; De La-Rocque et al., 2020).

Several studies showed that tau is found at the PM. Tau filaments are found at the PM of AD brain (Gray et al., 1987) and phosphorylated tau is accumulated in lipid rafts in the aged brain of AD mouse model (Kawarabayashi et al., 2004). Moreover, membrane lipids, such as phosphatidylcholine (PC), cholesterol, and sphingolipid, were reported to be associated with paired helical filament (PHF) purified from the brains of AD patients (Gellermann et al., 2006), further supporting the idea that tau can bind to membrane. The tau-membrane interaction is gaining growing attention since accumulating evidence has suggested that a majority of tau is secreted in a vesicle-free forms in both physiological and pathological conditions via unconventional translocation across the PM (reviewed in Brunello et al., 2020). Ectosomal secretion of tau is only found under physiological condition (Dujardin et al., 2014). In accordance with these findings, several studies showed that most of extracellular tau exists in vesicle-free forms, while only small fraction of tau is found in the vesicles (Karch et al., 2012; Plouffe et al., 2012; Simon et al., 2012; Pooler et al., 2013; Dujardin et al., 2014; Wegmann et al., 2016; Yan et al., 2016; Wang et al., 2017; Guix et al., 2018; Katsinelos et al., 2018; Merezhko et al., 2018). However, it is not clear to what extent each form of extracellular tau contributes to the propagation of tau pathology and neurodegeneration *in vivo*. Several mechanisms of the interaction between vesicle-free tau and the PM have been suggested, such as interactions through heparan sulfate proteoglycans (HSPGs) (Holmes et al., 2013; Rauch et al., 2018; Stopschinski et al., 2018; Hudak et al., 2019; Puangmalai et al., 2020), membrane bound muscarinic receptor (Morozova et al., 2019), LRP1 (Rauch et al., 2020), and direct binding to membrane lipids. In this review, we will review current understanding of the tau-membrane interaction focusing on the role of tau-binding molecules at the membrane and the consequences of the tau-membrane interaction on tau aggregation and neurotoxicity.

## Membrane Binding Domains of Tau

**FIGURE 1:**
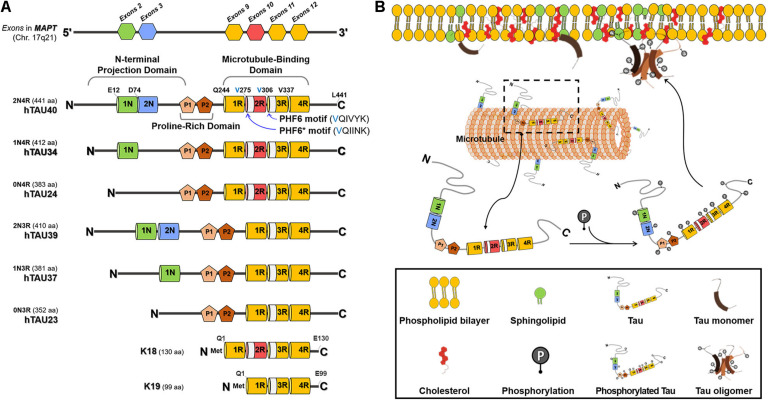
Structure and membrane interaction of tau. **(A)** The isoforms of human tau. Tau is encoded by *MAPT* gene. Tau is mainly found as 6 isoforms in the adult human brain. Tau isoforms contain 0, 1 or 2 of N-terminal inserts (0N, 1N, or 2N) and 3 or 4 of C-terminal microtubule (MT)-binding-repeats (3R or 4R). The longest 2N4R tau (commonly referred to as tau40) consists of three functional domains including N-terminal acidic projection domain, proline-rich domain, and C-terminal MT-binding domain (MTBD). 2R- and 3R-region contain PHF6*- and PHF6-motif, respectively. K18 and K19 tau fragments are the MTBD fragments from 4R and 3R tau, respectively. **(B)** Schematic diagram of the tau-membrane interaction. Tau is a natively unstructured MT-associated protein that plays important roles in regulating microtubule dynamics, cellular trafficking, and signaling pathways. Tau undergoes a variety of post-translational modifications and conformational changes, leading to accumulation of tau aggregates, such as toxic oligomers and fibrils, under pathological conditions. Membrane lipids, such as sphingolipids and cholesterol, play critical roles in the tau-membrane interaction. The tau-membrane interaction may facilitate tau fibrillization through providing a platform favorable for tau nucleation.

Human tau is encoded by the microtubule-associated protein tau gene (*MAPT*) which has 16 exons. Tau is mainly found as 6 isoforms in neurons of the adult human brain, which are generated by alternative splicing of exon 2, exon 3, and exon 10 (Figure 1A; Wang and Mandelkow, 2016). Tau isoforms contain 0, 1, or 2 of N-terminal inserts (0N, 1N, or 2N) and 3 or 4 of C-terminal MT-binding-repeats (3R or 4R). The longest 2N4R tau (commonly referred to as tau40) consists of three functional domains including N-terminal acidic projection domain, proline-rich domain, and C-terminal MT-binding domain (MTBD) (Figure 1A; Barre and Eliezer, 2006; Simic et al., 2016). Among them, the MTBD of tau plays critical roles in MT binding and aggregation (reviewed in Brunello et al., 2020), while the N-terminal acidic projection domain regulates MT binding affinity (Matsumoto et al., 2015) and the proline-rich domain mediates the interaction of tau with other proteins with SRC homology 3 (SH3) domains including Fyn kinase (Lee et al., 1998). The MTBD of tau has two hexapeptide motifs, PHF6 motif (VQIVYK) in all tau isoforms and PHF6^∗^ motif (VQIINK) only in 4R tau isoforms, which are fundamental for tau aggregation (von Bergen et al., 2000, 2001; Seidler et al., 2018). K18 and K19 tau fragments are the MTBD fragments from 4R and 3R tau, respectively. Given the importance of the MTBD in tau functions, K18 and K19 tau fragments have been used in many experiments instead of full-length tau.

Besides the aggregation and interaction with MT, the MTBD of tau is also involved in the tau-membrane interaction. Full-length 2N4R tau, K18, and K19 are all able to bind to lipid vesicles *in vitro* with an 2N4R tau > K18 > K19 rank potency order, suggesting that in addition to MTBD domain, additional regions may participate in the tau-membrane interaction and within MTBD domain, 2R region is important for the tau-membrane affinity (Barre and Eliezer, 2006; Kunze et al., 2012; Georgieva et al., 2014; Ait-Bouziad et al., 2017). In particular, two hexapeptide motifs were demonstrated to play an important role in the formation of the tau-phospholipid complexes, which are easily converted to elongated fibrils in acidic pH and toxic to primary hippocampal neurons (Ait-Bouziad et al., 2017; Fanni et al., 2019). Another study showed that the N-terminal projection domain of tau is also involved in the interaction of tau with the cytoplasmic face of the axonal membrane (Brandt et al., 1995; Arrasate et al., 2000). In PC12 cells stably expressing rat tau, both full-length and N-terminal fragment of tau are detected in the PM demonstrated by immunoelectron microscopy and subcellular fractionation (Brandt et al., 1995). It was further confirmed that tau is detected in the membrane fraction and N-terminal fragment of tau containing proline-rich domain is important for the tau-membrane association in COS-1 cells (Arrasate et al., 2000). Finally, Mari et al. (2018) suggested that the MTBD of tau is involved in the binding of tau to lipid membranes made from total brain lipid extracts and N-terminal region of tau promotes tau aggregation once tau binds to membrane. Taken together, both N-terminal projection and MTBD contribute to the tau-membrane interaction.

## The Interaction of Tau With Membrane Proteins

Accumulating evidence demonstrated that tau interacts with the membrane through binding to several different membrane proteins and thereby induce cellular dysfunctions and propagation of tauopathies. Extracellular tau is known to induce cytotoxicity by increasing intracellular calcium levels through muscarinic cholinergic receptor (Gomez-Ramos et al., 2006, 2008; Kanekiyo and Bu, 2014; Zollo et al., 2017). The administration of recombinant tau protein into the culture media induces intracellular calcium release in SH-SY5Y cells, COS-7 cells transiently expressing muscarinic receptor, and primary neurons (Gomez-Ramos et al., 2008). Muscarinic receptor subtype M1 and M3 mainly mediate tau-induced calcium disturbance in COS-7 cells and C-terminal region of tau is involved in the interaction with muscarinic receptor (Gomez-Ramos et al., 2008). Morozova et al. (2019) also demonstrated that phosphomimetic recombinant tau and tau purified from AD brains induce neurotoxicity through muscarinic receptor subtype M1 and M3-dependent uptake in primary neurons and mouse hippocampus.

Shrivastava et al. (2019) identified 29 intrinsic neuronal membrane proteins which interact with preformed 1N3R tau fibrils by proteomic screening using nanoLC-MS/MS after immunoprecipitating biotinylated tau fibrils in primary neurons. Among identified interaction partners, α3 subunit of Na^+^/K^+^-ATPase (NKA) and GluA2 subunit of α-amino-3-hydroxy-5-methyl-4-isoxazolepropionic acid (AMPA) receptor strongly interact with 1N3R tau fibrils, whereas GluN1 and GluN2B subunits of N-methyl-D-aspartic acid (NMDA) receptor only weakly interact with tau fibrils. Clustering of tau fibrils at excitatory synapses redistributes and reduces α3-NKA and increases GluA2-AMPA receptors at the synapse, leading to higher vulnerability to 4-aminopridine induced neuronal activity (Shrivastava et al., 2019).

Recently, low-density lipoprotein receptor related protein 1 (LRP1), a member of the low-density lipoprotein receptor (LDLR) family (Herz et al., 1988), was identified as a receptor for extracellular tau by genetic screening using CRISPR interference technology (Rauch et al., 2020). Silencing of LRP1 completely inhibits the uptake of all 6 full-length tau isoforms with 0N, 1N, or 2N and 3R or 4R as well as K18 and K19 fragments, suggesting that MTBD is the potential interaction site in H4 cells (Rauch et al., 2020). Lysine residues in MTBD and N-terminus of tau are demonstrated to be involved in the interaction with LRP1. Furthermore, knockdown of LRP1 in neurons dramatically suppresses tau spread across the brain in the mouse unilaterally injected with AAV expressing human tau with P301L mutant in hippocampus (Rauch et al., 2020), suggesting that LRP1 may represent a novel therapeutic target for tauopathies. Internalization of tau by LRP1 was further demonstrated using ^125^I-labeled recombinant 2N4R tau in LRP1-deficient CHO cells (Cooper et al., 2020). The direct binding of 2N4R tau monomer with LRP1 was confirmed utilizing surface plasmon resonance (SPR) analysis. In the SPR analysis, 2N4R tau isoform showed higher affinity for LRP1 than 2N3R tau isoform, suggesting that the R2 domain of tau is important for their binding (Cooper et al., 2020). In the same study, Cooper and colleagues showed that phosphorylation of tau reduces its affinity for LRP1 by comparing binding affinity of 2N4R tau isoform and its phosphomimetic form. They also identified Sortilin-related receptor (SORL1) as a tau receptor which is structurally similar to LDLR and physically interacts with LRP1 (Spoelgen et al., 2009; Dieckmann et al., 2010). The genetic mutation of SORL1 is known to be associated with late-onset AD (Kanekiyo and Bu, 2014; Zollo et al., 2017). As for LRP1, SORL1 increases tau seeding effect in HEK293 cells stably expressing the K18 (P301S) fluorescence resonance energy transfer (FRET) biosensor (Cooper et al., 2020).

In another studies, Annexin A2 and A6, calcium-dependent phospholipid-binding proteins, were identified as tau-binding partners using co-immunoprecipitation analysis (Gauthier-Kemper et al., 2011, 2018). The extreme N-terminal tau (1–44) encoded in the first exon of MAPT is sufficient for binding to annexin A2, whereas tau containing only the C-terminal (256–441) is not (Gauthier-Kemper et al., 2018). Tau and annexin A2 are colocalized at the tip of processes in PC12 cells and primary cortical neurons. Their interaction regulates the fluctuations in axon growth by trapping tau at the end of the process (Gauthier-Kemper et al., 2011).

Cellular prion protein (PrP^c^) is also demonstrated to interact with 2N4R or 0N3R tau. N-terminal residues (1–91) and repeat domain (186–283) of tau participate in its interaction with PrP^c^ (Han et al., 2006; Wang et al., 2008). Recently, it is reported that K18 tau fibrils also bind to PrP^c^ which mediates internalization of K18 tau fibrils in N2a cells (De Cecco et al., 2020). PrP^c^ is also known as a receptor for amyloid β (Aβ) and dysregulated in the brains of AD patients (Zhang et al., 2019). However, the clinical and therapeutical implications of PrP^c^ in tauopathies remain elusive.

Amyloid-beta precursor protein (APP) plays central roles in the pathogenesis of AD since it is precursor of Aβ and its mutations are associated with familial AD. Interestingly, several studies demonstrated that recombinant tau fibrils or tau from brain extract directly interact with C-terminal of APP (Smith et al., 1995; Giaccone et al., 1996; Islam and Levy, 1997). Furthermore, Takahashi et al. (2015) demonstrated that 1N4R and 1N3R tau fibrils, but not monomers, binds to APP through its N-terminal extracellular region and induces intracellular templated misfolding in SH-SY5Y cells, suggesting that R2 domain does not critically mediate the interaction of tau fibrils with APP. In this study, seed-based templated misfolding was intensively characterized by AT-8 immunostaining, western blot and immunoelectron microscopy in Sarkosyl-insoluble fractions. They did not find any effect of AD-associated mutations of APP on the seeding capacity of tau fibrils, suggesting that pathogenic mutations of APP do induce Aβ production but not promote tau propagation. In another study, Puzzo et al. (2017) demonstrated that 2N4R tau oligomer also binds to APP with Swedish mutation (APP_swe_) in HEK293 cells demonstrated by membrane fractionation followed by immunoprecipitation. Binding of tau with APP was barely observed in hippocampal neurons from APP KO mice compared to those from WT mice. They further demonstrated that impairments in LTP and memory caused by tau oligomers are dependent on the presence of APP in mice. APP is also reported to mediate the internalization of tau oligomer and thereby tau oligomer-induced disruption of Ca^2+^ signaling and Ca^2+^-dependent gliotransmitter release in primary astrocytes (Piacentini et al., 2017), suggesting that APP may be a critical player that regulates tau-induced toxicity and cellular dysfunctions in tauopathies.

CX3CL1/CX3CR1 axis contributes to phagocytosis of tau in microglia (Bolos et al., 2017). CX3CL1 (Fractalkine) is a ligand for chemokine receptor CX3CR1. Multiple lines of evidence suggest that CX3CL1/CX3CR1 axis plays important roles in the neuropathology of various brain disorders such as cerebral ischemia, epilepsy, and AD (Reviewed in Pawelec et al., 2020). Bolos et al. (2017) reported that CX3CR1 deficiency decreases the internalization of 2N4R tau monomer in microglia both *in vitro* and *in vivo*. They proved that phosphorylation of tau at S396, which is strongly implicated in AD-associated tau pathology, reduces its affinity for CX3CR1 using affinity chromatography technology. Both phosphorylated tau at S396 levels and CX3CL1 expression are increased in advanced Braak stages of AD, raising a possibility that CX3CL1 may inhibits tau clearance by microglia through competing with tau for binding to CX3CR1 (Bolos et al., 2016).

So far, several different membrane proteins have been identified to interact with tau. As discussed earlier, both N-terminal projection and MTBD are in general critical for the interaction of tau with membrane proteins. However, C-terminal region of tau also contributes to its interaction with membrane depending on binding partners. Depending on tau species and cell types, interaction of tau with these membrane proteins leads to various consequences, such as cytotoxicity, disturbance of cellular signaling, defect of intrinsic functions of membrane proteins, dysfunction in neuronal activity and gliotransmitter release, and propagation of tau pathology. However, it needs to be further elucidated whether and how these membrane proteins interplay each other for the interaction with tau and pathological events. Moreover, it is also unanswered what the pathological and therapeutical implications of those in tauopathies are.

## The Interaction of Tau With Proteoglycans

Growing evidence suggests that HSPGs are involved in the tau-membrane interaction. HSPGs are ubiquitously expressed at the extracellular matrix and the PM, which consist of the core proteoglycan with one or more heparan sulfate (HS) chains (Iozzo, 1998; Sarrazin et al., 2011). Holmes et al. (2013) first demonstrated that HSPGs interact with extracellular tau fibrils, such as K18 and 2N4R tau fibrils, and thereby mediate their uptake via macropinocytosis in C17.2 cells as well as primary hippocampal neurons using immunofluorescence and electron microscopy analyses. Pharmacological- and enzymatic inhibition of HSPG using chlorate, heparin, and heparinase inhibit tau fibril uptake and templated misfolding in HEK293T tau FRET biosensor cell line in a dose-dependent manner. They further proved that HSPGs mediate the internalization of 2N4R tau fibrils in neurons of mouse brains. Later, same group reported that tau trimer is the minimal seed unit to induce templated misfolding in a HSPG-dependent manner using a split-luciferase assay with tau RD (P301S) (Mirbaha et al., 2015). Interestingly, HSPG-mediated uptake pathway regulates internalization of brain-derived tau oligomers from AD and dementia with Lewy bodies, whereas alternative pathways are involved in the internalization of tau oligomers from progressive supranuclear palsy (Puangmalai et al., 2020). Moreover, it was shown that the syndecan, a transmembrane type of HSPG, also contributes to uptake of tau fibrils via lipid-raft-dependent endocytosis. Especially, overexpression of CDC3, a neuron predominant syndecan, increased 2N4R tau fibril uptake. Inhibition of several genes involved in HSPG synthesis strongly inhibits the internalization of tau monomer, oligomer, and fibril in cell cultures (Rauch et al., 2018; Stopschinski et al., 2018). Besides tau uptake, HSPGs mediate tau secretion. Two different groups demonstrated that inhibition of HSPGs using chlorate and heparinase suppresses tau secretion in different cells, such as N2a, SH-SY5Y, and primary neurons (Katsinelos et al., 2018; Merezhko et al., 2018). HS is composed of disaccharide repeats and glucosamine with sulfate moieties (Xu and Esko, 2014). The extended study identified that N-sulfation and 6-O-sulfation of HS are important for 2N4R tau fibril binding using a small heparin mimetic library (Stopschinski et al., 2018). It is further demonstrated that 6-O-sulfation mediates 2N4R tau monomer uptake in H4, induced pluripotent stem cell (iPSC)-derived neurons, and mouse hippocampal slice culture, while 2-O-sulfation does not (Rauch et al., 2018). In addition, the presence of 3-O-sulfation enhances binding of 2N4R tau to HS in SPR assay, and reduction of 3-O-sulfate decreases both cell surface binding and internalization of tau in HS 3-O-sulfotransferase-1-dificient cells (Zhao et al., 2020). Proline-rich domain 2 and R2 domain of 2N4R tau are identified as specific interaction sites for 3-O-sulfation by nuclear magnetic resonance (NMR) titrations mapping (Zhao et al., 2020). There is also conflicting finding. The internalization of monomeric tau is reported to be independent of HSPGs in primary astrocytes (Perea et al., 2019), implying that different tau species may interact with different partners at the PM and/or tau interacts with different partners in cells with different origins. Overall, HSPGs play a critical role in tau aggregation and intercellular transmission, suggesting that suppressing the interaction between tau and HSPGs could represent therapeutic target for tauopathies.

## The Role of Phosphorylation in the Interaction of Tau With Membrane

Tau undergoes a variety of PTMs, such as phosphorylation, glycosylation, acetylation, and truncation (Martin et al., 2011). Although the precise roles of PTMs in tau pathology remain elusive, hyperphosphorylation of tau is highly correlated with the formation of neurofibrillary tangles in AD (Martin et al., 2011). Phosphorylation of tau impairs the interaction of tau with MT, resulting in an increase of free tau eligible for aggregation (Martin et al., 2011).

The role of phosphorylation of tau in the tau-membrane interaction is debatable. Membrane-associated tau is reduced upon treatment of okadaic acid, a phosphatase inhibitor, in both COS-1 and PC12 cells expressing tau (Arrasate et al., 2000; Maas et al., 2000). Pooler et al. (2012) showed that tau in the membrane fraction from rat cortical neurons is primarily dephosphorylated, which is negative against PHF-1 (specific for phosphorylated Ser^396^/Ser^404^) and AT8 (specific phosphorylated Ser^202^/Thr^205^) antibodies. Their findings were further demonstrated in human neuronal SH-SY5Y (Ekinci and Shea, 2000) and rat neuronal PC12 cells (Maas et al., 2000). They also showed that inhibition of Casein kinase 1 and Glycogen Synthase Kinase-3β (GSK3β), well-established tau kinases, increases the association of tau with the neuronal PM, whereas inhibition of Protein phosphatase 2A decreases the membrane association of tau (Pooler et al., 2012). In addition, phosphorylation-mimicking 0N3R tau, in which several serine residues in the proline-rich and C-terminal regions are substituted with glutamate, is barely detected at the PM (Eidenmuller et al., 2001). Similarly, mimicking phosphorylation in the N-terminal half of tau including N-terminal projection domain and proline-rich domain significantly decreases the association of tau with membrane in CHO cells (Pooler et al., 2012). In mouse brain slice culture, the ratio of dephosphorylated tau to total tau is increased in the membrane fraction of 3×Tg-AD slice, which is increased in WT slice by neuronal activity but not in 3×Tg-AD slice (Croft et al., 2017).

On the contrary, there is also conflicting evidence to support positive effects of phosphorylation on the tau-membrane interaction. Phosphorylated high molecular weight tau is increased in the PM fraction during apoptosis in the PC12 cells (Shelton and Johnson, 2001). In AD mouse model, Aβ accumulation in lipid rafts leads to the recruitment of apolipoprotein E (APOE) and phosphorylated tau to the membrane in an age-dependent manner (Kawarabayashi et al., 2004). Another study demonstrated that tyrosine phosphorylation of tau by Fyn kinase increases the association of 2N4R tau with Fyn and thereby tau localization in detergent-resistant microdomain of membrane in primary neurons (Usardi et al., 2011). In mouse neuronal N2a cells expressing human 0N4R tau isoform, phosphorylated oligomeric tau is predominantly localized in the PM (Merezhko et al., 2018) and secreted across the PM through a vesicle-free pathway (Katsinelos et al., 2018). Moreover, tau phosphorylation by GSK3β does not affect 1N4R tau monomer-membrane binding but enhances tau oligomer-membrane binding in small unilamellar lipid vesicles which consist of 1-palmitoyl-2-oleoylphosphatidylcholine (POPC) or di-palmitoylphosphatidylcholine (DPPC) (Nuebling et al., 2020).

These contradictory data may be because each study used different tau species and cell lines and addressed different phosphorylation sites. Although the results across the studies apparently seem to be conflicting, it is speculative that phosphorylation of physiological tau may inhibit the tau-membrane interaction, whereas phosphorylation of pathological tau oligomers and fibrils may promote the tau-membrane interaction (Figure 1B).

## The Role of Lipids in the Membrane Interaction and Fibrillization of Tau

As aforementioned, tau is a natively highly soluble unstructured protein under physiological conditions (Jeganathan et al., 2008; Mukrasch et al., 2009). Under pathological conditions, tau undergoes a variety of PTMs and conformational changes, leading to pathological tau aggregation (Wang and Mandelkow, 2016). Although the precise mechanisms of structural transition and aggregation of tau are not fully understood yet, numerous factors, such as mutations, phosphorylation, acetylation, proteolysis, oxidative stress, and membrane lipids, have been suggested to be able to facilitate tau fibrillization (Martin et al., 2011). Here, we summarize the most recent understanding of the tau-membrane interaction and its role in tau aggregation.

It is well known that polyanionic cofactors, such as heparin (Friedhoff et al., 1998), polyglutamate (Friedhoff et al., 1998), RNA (Kampers et al., 1996), arachidonic acid (King et al., 2000), and congo red (Bandyopadhyay et al., 2007), facilitate fibrillization of tau. The PM anchored chimeric tau proteins capture the 2N4R tau and trigger the assembly of tau into β-sheet fibrils in cell culture detected by thiazine red (Campos-Pena et al., 2009; Lira-De León et al., 2009). Given that the PM contains anionic lipids, it is not surprising that the PM can trigger tau fibrillization. In line with these results, numerous studies have shown that various membrane lipids interact with tau and regulate its fibrillization in the experimental settings with PM-mimicking lipid vesicles (Table 1). When 2N4R monomeric tau is incubated with anionic PS liposome, at least 15 % of all filaments are associated with vesicles, which appear to be expanded on the vesicle surface observed by transmission electron microscopy (TEM) (Chirita et al., 2003). The large unilamellar vesicles (LUV), consisting of PS/PC, induce aggregation of K18 monomeric tau likely by providing a platform for charge neutralization and thereby facilitating protein-protein interaction demonstrated by fluorescence correlation spectroscopy (FCS) and thioflavin T assay (Elbaum-Garfinkle et al., 2010). In LUV with POPC and 1-palmitoyl-2-oleoyl-sn-glycero-3-phospho-L-serine (POPS) mixtures, K19 monomeric tau binds to membrane by electrostatic forces between the positive charge in tau and the negative charge in phospholipid headgroups, which is predominantly structured to β-sheet-like intermediates evidenced by circular dichroism (CD) spectra and solid-state NMR analysis (Kunze et al., 2012). In small unilamellar vesicles (SUV) only with negatively charged POPS, K19 monomeric tau is converted to a α-helix structure, suggesting that the structural change of K19 fibril depends on the extent of membrane charge (Kunze et al., 2012). Another study showed that the interaction of K19 monomeric tau with membrane is mediated by short amphipathic helices formed in the membrane-bound state (Georgieva et al., 2014). Recombinant 2N4R monomeric tau preferentially interacts with anionic 1,2-dimyristoyl-sn-glycero-3-[phospho-rac-(1-glycerol)] (DMPG) monolayer compared to zwitterionic 1,2-dimyristoyl-sn-glycero-3-phosphocholine (DMPC), further supporting that anionic charge facilitates the tau-membrane interaction (Jones et al., 2012). Insertion of 2N4R monomeric tau into the DMPG mono- and bilayer results in compaction of tau structure, disruption of lipid packing, and compromising membrane structural integrity demonstrated by X-ray reflectivity (Jones et al., 2012). K18 monomer and hyperphosphorylation-mimicking 2N4R monomeric tau are more potent in membrane-templated misfolding and assembly into β-sheet-rich aggregates than native 2N4R monomeric tau (Majewski et al., 2020). In the MTBD, two hydrophobic hexapeptides motifs, PHF6^∗^- and PHF6-motif, are reported to be a core of K18/phospholipid complex and PHF6 motif has a higher propensity to form β-strand-like structure than PHF6^∗^ motif (Barre and Eliezer, 2013; Ait-Bouziad et al., 2017). In addition, a study using CD spectra and X-ray reflection revealed that acetylation of PHF6 motif facilitates membrane-templated fibrillization and membrane destabilization at the DMPG monolayer and LUV of POPC/POPG (Fanni et al., 2019). So far, it is not clear what factors regulate the tau-membrane interaction and fibrillization on the membrane. Although tau can initiate self-assembly without additional factors on the lipid membrane, it is recently reported that sodium triggers the membrane interaction and fibrillization of 2N4R tau at the supported lipid membrane (SLM) with total lipid extract from porcine brain, whereas potassium inhibits these processes (Mari et al., 2018). N-terminal region of tau promotes self-assembly and growth of tau fibrils on the SLM (Mari et al., 2018). Further studies are warranted to identify the regulators of the tau-membrane interaction and fibrillization in live cells.

**TABLE 1:** Summary of evidence supporting conformational change of Tau at the membrane.

Tau				Membrane		Detection	References
Source	Isoform	Mutant	Species	Component	Type		
Human	2N4R		monomer	PS	Liposome	TEM	Chirita et al., 2003
				C_1__4_E_8_/C_2__0_H_4__1_SO_4_Na	Micelle	Ultracentrifuge	
Human	2N4R		TF	COS-7 cells	PM	Thiazine red	Campos-Pena et al., 2009; Lira-De León et al., 2009
Human	2N4R(Δ392-441)		TF				
Human	PHF		TF				
Human	K18		monomer	PS/PC	LUV	FCS	Elbaum-Garfinkle et al., 2010
Human	K18-Alexa 488		monomer			Thioflavin T	
Human	2N4R		monomer	DMPG	Monolayer	X-ray	Jones et al., 2012
				DMPG	Bilayer		
Human	K19		monomer	DMPS	SUV	CD spectra	Kunze et al., 2012
				DMPC/DMPS	SUV, LUV	Thioflavin T	
				POPC/POPS	LUV	(Thioflavin T negative)	
				SDS	Micelle	solid-state NMR	
Human	K18		monomer	SDS	Micelles	NMR	Barre and Eliezer, 2013
Human	0N3R		monomer	POPC/POPS	SUV	CW-ESR	Georgieva et al., 2014
Human	K19		monomer	LPPG	Micelle		
				SDS	Micelle		
Human	K18		monomer	Porcine brain PS	Vesicle	NMR	Ait-Bouziad et al., 2017
Human	2N4R			Total lipid extract of porcine brain	SLM	AFM topography	Mari et al., 2018
Human	K18						
Human	N-Terminal Tau (a.a. 1-257)						
Human	Ac-PHF6		fibrils	DMPG	Monolayer	CD spectra	Fanni et al., 2019
Human	NH_3_^+^-PHF6		fibrils	POPC/POPG	LUV	X-ray	
Human	K18		monomer	DMPG	Monolayer	synchrotron GIXD	Majewski et al., 2020
Human	2N4R/3Epi	HMM	monomer				
Human	2N4R		monomer				
Human	0N4R	P301S	fibrils	DiPhPC	Bilayer	Thioflavin T	Esteras et al., 2020

*AFM, atomic force microscopy; CD, circular dichroism; CW-ESR, continuous-wave electron spin resonance, DiPhPC, 1,2-diphytanoyl 3-phosphocholine, FCS, Fluorescence correlation spectroscopy, LPPG, lyso-1-palmitoyl-phosphatidylglycerol; LUV, Large unilamellar vesicle; DMPC, 1,2-dimyristoyl-sn-glycero-3-phosphocholine; DMPG, 1,2-dimyristoyl-sn-glycero-3-phospho(1′-rac-glycerol); DMPS, 2,3-Dimercapto-1-propanesulfonic acid; HMM, hyperphosphorylation-mimicking mutant; NMR, nuclear magnetic resonance; PC, phosphatidylcholine; PM, Plasma Membrane; POPC, 1,2-dioleoyl-sn-glycero-3-phosphocholine; POPS, 1-palmitoyl-2-oleoyl-sn-glycero-3-phospho-l-serine; PS, phosphatidylserine; SDS, sodium dodecyl sulfate; SLM, Supported lipid membrane; SUV, Small unilamellar vesicle; TEM, Transmission electron microscopy; TF, Transfection.*

In the cellular contexts, tau interacts with the cytoplasmic face of the axonal membrane (Brandt et al., 1995; Arrasate et al., 2000). Tau R406W mutation reduces the interaction of 0N3R tau with a calcium-regulated PM–binding protein annexin A2, thereby inhibiting the binding of tau to the PM which results in increased fluctuations in axonal growth (Gauthier-Kemper et al., 2011). In oligodendrocytes, tau is localized at the ends of the cellular processes along with myelin basic protein (LoPresti et al., 1995). Moreover, tau mediates the regulation of process outgrowth by Fyn kinase through bridging the interaction between Fyn kinase and MT in oligodendrocytes (Klein et al., 2002). Recently, it is demonstrated that the binding of tau to membrane induces neurotoxicity (Ait-Bouziad et al., 2017; Esteras et al., 2020). The tau-phospholipid complexes are recognized by MC-1 and further converted into long tubular and filament-like aggregates under slightly acidic pH, suggesting that they have pathological properties (Ait-Bouziad et al., 2017). The tau-phospholipid complexes can be taken up by primary hippocampal neurons via endocytosis and induce cytotoxicity (Ait-Bouziad et al., 2017). Moreover, heparin-induced insoluble aggregates of 0N4R fibrillar tau interact with DiPhPC bilayer and alter the membrane conductance (Esteras et al., 2020). These changes in membrane conductance increase Ca^2+^ influx through opening voltage-gated calcium channel and sequentially reactive oxygen species levels via activating NADPH oxidase, eventually leading to cell death in both primary cortical neurons and astrocytes (Esteras et al., 2020). Hyperphosphorylated tau is also detected at both pre-and post-synaptic compartments in synaptosomes isolated from AD patients (Fein et al., 2008; Henkins et al., 2012; Tai et al., 2012). Recent study showed that pathological hyperphosphorylated tau but not physiological tau binds to synaptic vesicle at presynaptic terminals via N-terminal domain and interferes presynaptic functions, such as mobility and release of synaptic vesicles (Zhou et al., 2017). Two recent studies demonstrated that tau is secreted from the cells by direct translocation across the PM (Katsinelos et al., 2018; Merezhko et al., 2018). Katsinelos et al. (2018) demonstrated that 0N4R tau is recruited to the cytosolic leaflet of the PM by interacting with phosphatidyl inositol 4,5 phosphate [PI(4,5)P_2_] evidenced by interaction of tau with lipid strip and LUV containing PI(4,5)P_2_. In another study, Merezhko et al. (2018) demonstrated that tau is mainly located at the cytosolic leaflet of the PM and nuclear membrane in N2a cells expressing human 0N4R tau isoform. They further demonstrated that depletion of membrane cholesterol using methyl-β-cyclodextrin and sphingomyelin using sphingomyelinase or myriocin significantly inhibit tau secretion (Merezhko et al., 2018). Moreover, tau secretion is also suppressed by treating cells with docosahexaenoic acid (DHA) (Merezhko et al., 2018). DHA is a polyunsaturated fatty acid which is known to reduce membrane order and disrupt lipid raft microdomain. Interestingly, paired helical filaments purified from AD brains were shown to contain small amounts of cholesterol, phosphatidylcholine, and sphingolipids (Gellermann et al., 2006). Moreover, a pathogenic form of tau phosphorylated at S422 is found to be localized at flotillin 1-positive lipid raft regions of neuronal membrane in the brain of AD mouse model expressing APP_swe_ and PSEN2 together with tau P301L (Collin et al., 2014). However, it is not addressed whether tau directly binds to cholesterol, sphingolipids, or DHA in the cellular contexts. A recent study suggested another possibility of cholesterol engagement in the interaction of protein with the membrane. Doktorova et al. (2017) demonstrated that the presence of cholesterol in the membrane enhances the interaction of retroviral structural protein Gag to the phosphatidylserine-rich clusters of the membrane by increasing lipid packing and membrane surface charge density. Taken together, these findings suggest that cholesterol and sphingomyelin-dependent membrane microdomains play a critical role in tau secretion by mediating recruitment of tau to the cytosolic leaflet of the PM. By immunoelectron and immunofluorescence microscopy, Merezhko et al. (2018) demonstrated that tau located at the cytosolic leaflet of the PM is mainly oligomeric forms positive with AT8- and PHF13-specific phosphorylations but negative with thioflavin-S staining. They further demonstrated that inhibition of tau oligomerization and aggregation suppresses tau secretion. Katsinelos et al. (2018) demonstrated that tau secretion depends on the levels of phosphorylation rather than the site-specific phosphorylation, suggesting that pathogenic hyperphosphorylation and conformational change of tau are critical for membrane interaction and secretion of tau. Furthermore, Katsinelos et al. (2018) demonstrated that monomeric tau can disrupt the membrane integrity in a PI(4,5)P_2_-dependent manner in liposome. Indeed, tau has capable of disrupting the endosomal membrane, although the underlying mechanisms have not been fully elucidated (reviewed in Brunello et al., 2020). Taken together, all these data strongly suggest that lipids and proteoglycans in the membrane could play vital roles in the pathogenesis of tauopathies by mediating multiple different processes in tau propagation, such as aggregation, recruitment to the PM, secretion across the PM from donor cells, uptake in recipient cells, and release from intracellular vesicles to initiate templated misfolding.

A series of studies *in vitro* as well as *in vivo* showed that tau interacts with the PM. Moreover, the tau-membrane interaction facilitates pathological aggregation of tau in the experimental settings with liposomes. Moreover, the tau-membrane interaction causes neurotoxicity in primary cultures *in vitro.* However, it is not clear whether oligomerization of tau is induced at the surface of the PM after monomeric tau interacts with the PM, or oligomeric or fibrillar tau species produced somewhere else preferentially bind to the PM in the cellular contexts. Furthermore, it needs to be elucidated whether there is a difference in the propensity and/or preference of different lipid species to the interaction with different species of tau, such as monomeric, oligomeric, and fibrillar tau and how several different lipids cooperate each other for the binding, aggregation, and intercellular propagation of tau. Whether and how the tau-membrane interaction contributes to the tau pathology and neurodegeneration in the brain *in vivo* also remain elusive.

## The Association of Tau With Intracellular Organelle Membrane

The MT-associated protein tau is a cytosolic protein that is essential for MT assembly (Weingarten et al., 1975) and stability (Drubin and Kirschner, 1986). The PTMs in tau protein regulate the affinity of tau for MT (Lindwall and Cole, 1984). Once tau is released from MT, chances for tau to interact with other cellular partners, such as proteins and organelle membranes, rise (Morris et al., 2011). Here, we will discuss the interaction of tau with intracellular organelle membranes and its implication in the regulation of organelle’s function.

Perreault et al. (2009) reported that the contacts between rough endoplasmic reticulum (RER) membranes and mitochondria are increased in the brains of AD patients and spinal cord motor neurons of JNPL3 mice expressing P301L tau, which is correlated with an increase of tau on the surface of RER membranes. The mitochondria-ER contracts (MERCs) are involved in many different cellular processes, such as lipid metabolism, mitochondrial dynamics, autophagy, calcium/redox signaling, and apoptosis (Csordas et al., 2018; Moltedo et al., 2019). However, it remains to be further elucidated whether tau accumulation in RER affects the functions of MERCs and thereby contributes to the diseases. Farah et al. (2006) showed that tau is found at the Golgi membrane in the rat brain as well as motor neurons of mouse spinal cord. As of tau found in RER, tau in Golgi fraction is hyperphosphorylated which is recognized by PHF-1 and AT8 antibodies. However, whether tau in Golgi membrane affects the function of Golgi was not addressed. Wang et al. (2009) showed that when K18 tau with the ΔK280 mutant (Tau_RD_ΔK) is expressed in N2a cells, Tau_RD_ΔK interacts with LAMP-2A in lysosome and is thereby processed to highly amyloidogenic fragments which nucleate aggregation of tau. Growing evidence suggests that mitochondrial dysfunction is closely related to the pathophysiology of tauopathies (Szabo et al., 2020; Wang et al., 2020). Phosphorylated tau is also found at the mitochondria membrane (Jancsik et al., 1989; Rendon et al., 1990). Camilleri et al. (2013) demonstrated that 2N4R tau oligomers, but not monomers, induce membrane permeabilization in mitochondria-mimicking liposomes and trigger cytochrome c release from mitochondria isolated from SH-SY5Y cells. In a subsequent study, same group demonstrated that cardiolipin, a phospholipid unique to mitochondrial membrane, plays a critical role in mediating the interaction of 1N4R tau oligomers with mitochondria, leading to mitochondrial damage (Camilleri et al., 2020). They further provided the evidence that 10-N-nonyl acridine orange (NAO), a cardiolipin-specific dye, efficiently prevents tau oligomer-induced mitochondrial damages, such as swelling and cytochrome c release from mitochondria isolated from SH-SY5Y cells, raising a possibility that modulating cardiolipin may represent a novel therapeutic strategy for tauopathies (Camilleri et al., 2020). Phosphorylated tau also interacts with nuclear membrane through binding to nucleoporin 98 (Nup98) in tau-overexpressing transgenic mice and human AD brain tissue (Eftekharzadeh et al., 2018). Of note, abnormal binding of phosphorylated tau to Nup98 in nuclear membrane triggers mislocalization of Nup98 from the nuclear membrane to cytoplasm, which accelerates the aggregation of phosphorylated tau in cytoplasm (Eftekharzadeh et al., 2018). Despite a handful of studies showed the interaction of tau with intracellular organelles, whether and how the interaction of tau with organelle membrane triggers organelle dysfunction and contributes to tau fibrillization and pathology are largely unknown.

## Pathological Implications of Lipid Dysregulation in Tauopathies

Dysregulation of lipid metabolism has been under intense scrutiny in the pathogenesis of AD ever since the ε4 allele of APOE, a major lipoprotein in the brain, is identified as the strongest risk factor for AD (Kim et al., 2009). Moreover, epidemiological studies strongly suggested that conditions linked to lipid dysregulation, such as hypercholesterolemia, atherosclerosis, hypertension, and diabetes, increase the risk for AD. Accumulating evidence consistently demonstrated that several lipids, such as cholesterol, cholesterol ester, and sphingolipids, are accumulated in the brains of AD patients and AD mouse models (Cutler et al., 2004; Shobab et al., 2005; Di Paolo and Kim, 2011; Chan et al., 2012; Lazar et al., 2013; Tajima et al., 2013; Walter and van Echten-Deckert, 2013). AD is characterized by pathological hallmarks, such as abnormal accumulation of extracellular Aβ deposits and intracellular tau aggregates. It has been hypothesized that abnormal accumulation of Aβ initiates pathological cascades leading to accumulation of hyperphosphorylated tau, neuroinflammation, and neurodegeneration. Although the role of lipid metabolism on tauopathy is relatively less characterized compared to Aβ pathology, a growing evidence suggests that there are mutual regulations between lipid metabolism and tauopathy. Several groups showed that tangle-bearing neurons contain higher levels of unesterified cholesterol than tangle-free neighboring neurons in the brains of AD patients and transgenic mice expressing FTDP-17-associated P301L tau mutant as well as Niemann-Pick disease type C (NPC) (Giaccone et al., 1996; Distl et al., 2001, 2003; Girardot et al., 2003; Glockner and Ohm, 2014). Cholesterol accumulation due to the defective cholesterol trafficking is closely associated with the pathogenesis of NPC which develops tau pathology with neurofibrillary tangles. High cholesterol diets increase tau phosphorylation and promotes tau pathology in mouse and rabbit (Rahman et al., 2005; Ghribi et al., 2006; Glockner et al., 2011; Bhat and Thirumangalakudi, 2013). In addition, pharmacological and genetic induction of CYP46A1, an enzyme that catalyzes conversion of cholesterol to 24S-hydroxycholesterol, attenuates tau-associated pathologies in iPSC-derived AD neurons (van der Kant et al., 2019) and Tau22 mice expressing 1N4R human tau with G272V and P301S mutations (Burlot et al., 2015), respectively, whereas inhibition of CYP46A1 increases tau phosphorylation and Aβ-associated pathologies in APP23 mice expressing APP_swe_ mutant (Djelti et al., 2015). Of note, an increase of membrane cholesterol in primary neurons induces AD-like phenotypes, such as vesicular trafficking defect and increases of Aβ_42_ secretion, Aβ-mediated toxicity, and Aβ-induced tau truncation by calpain in a NMDA receptor-dependent manner (Nicholson and Ferreira, 2009; Nicholson et al., 2011; Marquer et al., 2014). Besides lipids, the proteoglycan levels and expression of enzymes participating in generation of sulfated glycosaminoglycans have been reported to be altered in AD brains (Wray and Noble, 2009; Ariga et al., 2010; Wang and Ding, 2014; Sepulveda-Diaz et al., 2015). Taken together, dysregulation of lipid metabolism and proteoglycans is unequivocally involved in the pathogenesis of human neurological disorders with tauopathies. However, it remains to be elucidated whether and how alteration of lipids and proteoglycans regulates the pathogeneses of tauopathies in *in vivo* experimental settings and human pathologies. Clinical implications of modulating tau-interacting molecules also need to be further elucidated.

## Conclusion and Future Perspectives

Elucidating the roles of the tau-membrane interaction are critical for understanding of the mechanisms of tau fibrillization, intercellular transmission, and dysfunction of biological processes associated with tauopathies. In this review, we have highlighted recent understanding of the tau-membrane interaction and its roles in pathophysiology. However, there is a significant gap in our knowledge of the mechanisms and roles of the tau-membrane interaction. The findings from cell-free systems and cell culture experiments have provided valuable mechanistical hints how tau-interacting molecules, such as lipids, proteoglycans, and proteins, regulate the tau-membrane interaction, tau aggregation, tau-induced neurotoxicity, and propagation of tau. It remains unanswered to what extent each tau-interacting molecule is involved in tauopathies and how they cooperate each other to mediate tau-associated pathological events. Numerous epidemiological studies and animal studies have shown a clear association between lipid dysregulation and tauopathies. However, it remains elusive whether the alteration of tau-interacting molecules could be causative factors for tauopathies and whether modulation of tau-interacting molecules could have therapeutic implications.

Different pathological tau species may interact with membranes of different origins which may induce deficits in many biological functions in a species-dependent manner. It should be kept in mind that tau also mediates several normal biological processes through interacting with membranes under physiological condition. Lipid compositions are critical for many biological processes, such as endocytosis, phagocytosis, secretion of various molecules, cellular signaling pathways, receptor-ligand interaction, synaptic transmission, and neuronal activity. So far, it has not been thoroughly examined whether and how the interaction of tau with membrane could regulate those cellular functions. Another interesting question is that if membrane provides a platform of tau nucleation, why are pathological tau aggregates not accumulated under physiological condition? Indeed, hyperphosphorylated tau oligomers but not fibrils are found at the cytosolic face of the PM in mouse neuronal cells under physiological condition (Zhou et al., 2017; Merezhko et al., 2018). This might be because (1) free tau exists at a subthreshold level for fibrillization due to trapping by MT, (2) pathological tau fibrils are quickly removed, (3) lipid compositions and characteristics of membrane are not favorable for pathological transitions of tau under physiological condition. It is also conceivable that there are unidentified factors that promotes tau fibrillization under pathological conditions or inhibits tau fibrillization under physiological conditions. Data collected so far raise another question. Where does tau initiate to oligomerize and aggregate? Since tau can interact with membranes of several different intracellular organelles as well as the PM, it would be interesting to answer whether these organelles are somehow involved in tau aggregation. Both monomeric- and aggregated tau are detected in extracellular milieu. It seems that monomeric tau secretion is physiological event. However, it is not clear what is the physiological role of monomeric tau secretion and whether and how the tau-membrane interaction induces pathological tau aggregation in extracellular space. Further studies are warranted to elucidate the precise mechanisms and the roles of the tau-membrane interaction in the pathophysiology of tauopathies. Comprehensive understanding of the nature and pathological role of the tau-membrane interaction will have critical implications for the development of novel therapeutic interventions for tauopathies.

## Author Contributions

EB, EL, and JK reviewed the literature and wrote the review. B-RL, JML, CJY, and EML contributed to the critical discussion and revision of the manuscript. EB and EL prepared the graphic and table. JK supervised the work. All authors contributed to the article and approved the submitted version.

## Conflict of Interest

The authors declare that the research was conducted in the absence of any commercial or financial relationships that could be construed as a potential conflict of interest.

## Abbreviations

A β: amyloid beta
AD: Alzheimer’s disease
AMPA: α-amino -3-hydroxy-5-methyl-4-isoxazolepropionic acid
APOE: apolipoprotein E
APP: Amyloid-beta precursor protein
APPswe: APP with Swedish mutation
CD: circular dichroism
DHA: docosahexaenoic acid
DMPC: 1,2-dimyristoyl-sn-glycero-3-phosphocholine
DMPG: 1,2-dimyristoyl-sn-glycero-3-phospho(1’-rac-glycerol)
DMPS: 2,3-Dimercapto-1-propanesulfonic acid
DPPC: di-palmitoylphosphatidylcholine
FCS: fluorescence correlation spectroscopy
GSK3 β: glycogen Synthase Kinase-3 β
HS: heparan sulfate
HSPG: heparan sulfate proteoglycan
iPSC: induced pluripotent stem cell
LDLR: low-density lipoprotein receptor
LRP1: lipoprotein receptor related protein 1
LUV: large unilamellar vesicle
NMDA: N-methyl -D-aspartic acid
MAPT: microtubule-associated protein tau gene
MERC: mitochondria-ER contracts
MT: microtubule
MTBD: microtubule-binding domain
NAO: 10-N-nonyl acridine orange
NKA: Na^+^/K^+^-ATPase
NMR: nuclear magnetic resonance
NPC: Niemann-Pick disease type C
Nup98: nucleoporin 98
PC: phosphatidylcholine
PHF: paired helical filament
PI(4,5)P_2_: phosphatidyl inositol 4,5 phosphate
PM: plasma membrane
POPC: 1-palmitoyl-2-oleoylphosphatidylcholine
POPS: 1-palmitoyl-2-oleoyl-sn-glycero-3-phospho-l-serine
PrP^c^: Cellular prion protein
PTM: post-translational modification
RER: rough endoplasmic reticulum
SH3: SRC homology 3
SLM: supported lipid membrane
SORL1: Sortilin-related receptor
SPR: surface plasmon resonance
SUV: small unilamellar vesicles
TauRD Δ K: Tau with the Δ K280 mutant
TEM: transmission electron microscopy.

## References

[B1] Ait-Bouziad N. Lv G. Mahul-Mellier A. L. Xiao S. Zorludemir G. Eliezer D. (2017). Discovery and characterization of stable and toxic Tau/phospholipid oligomeric complexes. *Nat. Commun.* 8:1678. 10.1038/s41467-017-01575-4 29162800 PMC5698329

[B2] Ariga T. Miyatake T. Yu R. K. (2010). Role of proteoglycans and glycosaminoglycans in the pathogenesis of Alzheimer’s disease and related disorders: amyloidogenesis and therapeutic strategies–a review. *J. Neurosci. Res.* 88 2303–2315. 10.1002/jnr.22393 20623617

[B3] Arrasate M. Perez M. Avila J. (2000). Tau dephosphorylation at tau-1 site correlates with its association to cell membrane. *Neurochem. Res.* 25 43–50. 10.1023/a:1007583214722 10685603

[B4] Bandyopadhyay B. Li G. Yin H. Kuret J. (2007). Tau aggregation and toxicity in a cell culture model of tauopathy. *J. Biol. Chem.* 282 16454–16464. 10.1074/jbc.M700192200 17428800

[B5] Barbier P. Zejneli O. Martinho M. Lasorsa A. Belle V. Smet-Nocca C. (2019). Role of tau as a microtubule-associated protein: structural and functional aspects. *Front. Aging Neurosci.* 11:204. 10.3389/fnagi.2019.00204 31447664 PMC6692637

[B6] Barre P. Eliezer D. (2006). Folding of the repeat domain of tau upon binding to lipid surfaces. *J. Mol. Biol.* 362 312–326. 10.1016/j.jmb.2006.07.018 16908029

[B7] Barre P. Eliezer D. (2013). Structural transitions in tau k18 on micelle binding suggest a hierarchy in the efficacy of individual microtubule-binding repeats in filament nucleation. *Protein Sci.* 22 1037–1048. 10.1002/pro.2290 23740819 PMC3832040

[B8] Bhat N. R. Thirumangalakudi L. (2013). Increased tau phosphorylation and impaired brain insulin/IGF signaling in mice fed a high fat/high cholesterol diet. *J. Alzheimers Dis.* 36 781–789. 10.3233/JAD-2012-121030 23703152 PMC4445975

[B9] Bolos M. Llorens-Martin M. Jurado-Arjona J. Hernandez F. Rabano A. Avila J. (2016). Direct evidence of internalization of tau by microglia in vitro and in vivo. *J. Alzheimers Dis.* 50 77–87. 10.3233/JAD-150704 26638867

[B10] Bolos M. Llorens-Martin M. Perea J. R. Jurado-Arjona J. Rabano A. Hernandez F. (2017). Absence of CX3CR1 impairs the internalization of Tau by microglia. *Mol. Neurodegener.* 12:59. 10.1186/s13024-017-0200-1 28810892 PMC5558740

[B11] Braak H. Braak E. (1991). Neuropathological stageing of Alzheimer-related changes. *Acta Neuropathol.* 82 239–259. 10.1007/BF00308809 1759558

[B12] Brandt R. Leger J. Lee G. (1995). Interaction of tau with the neural plasma membrane mediated by tau’s amino-terminal projection domain. *J. Cell Biol.* 131 1327–1340. 10.1083/jcb.131.5.1327 8522593 PMC2120645

[B13] Brelstaff J. Tolkovsky A. M. Ghetti B. Goedert M. Spillantini M. G. (2018). Living neurons with tau filaments aberrantly expose phosphatidylserine and are phagocytosed by microglia. *Cell Rep.* 24 1939–1948.e4. 10.1016/j.celrep.2018.07.072 30134156 PMC6161320

[B14] Brunello C. A. Merezhko M. Uronen R. L. Huttunen H. J. (2020). Mechanisms of secretion and spreading of pathological tau protein. *Cell. Mol. Life Sci.* 77 1721–1744. 10.1007/s00018-019-03349-1 31667556 PMC7190606

[B15] Burlot M. A. Braudeau J. Michaelsen-Preusse K. Potier B. Ayciriex S. Varin J. (2015). Cholesterol 24-hydroxylase defect is implicated in memory impairments associated with Alzheimer-like Tau pathology. *Hum. Mol. Genet.* 24 5965–5976. 10.1093/hmg/ddv268 26358780

[B16] Camilleri A. Ghio S. Caruana M. Weckbecker D. Schmidt F. Kamp F. (2020). Tau-induced mitochondrial membrane perturbation is dependent upon cardiolipin. *Biochim. Biophys. Acta* 1862:183064. 10.1016/j.bbamem.2019.183064 31521630

[B17] Camilleri A. Zarb C. Caruana M. Ostermeier U. Ghio S. Hogen T. (2013). Mitochondrial membrane permeabilisation by amyloid aggregates and protection by polyphenols. *Biochim. Biophys. Acta* 1828 2532–2543. 10.1016/j.bbamem.2013.06.026 23817009

[B18] Campos-Pena V. Tapia-Ramirez J. Sanchez-Torres C. Meraz-Rios M. A. (2009). Pathological-like assembly of tau induced by a paired helical filament core expressed at the plasma membrane. *J. Alzheimers Dis.* 18 919–933. 10.3233/JAD-2009-1198 19749435

[B19] Chan R. B. Oliveira T. G. Cortes E. P. Honig L. S. Duff K. E. Small S. A. (2012). Comparative lipidomic analysis of mouse and human brain with Alzheimer disease. *J. Biol. Chem.* 287 2678–2688. 10.1074/jbc.M111.274142 22134919 PMC3268426

[B20] Chirita C. N. Necula M. Kuret J. (2003). Anionic micelles and vesicles induce tau fibrillization in vitro. *J. Biol. Chem.* 278 25644–25650. 10.1074/jbc.M301663200 12730214

[B21] Collin L. Bohrmann B. Gopfert U. Oroszlan-Szovik K. Ozmen L. Gruninger F. (2014). Neuronal uptake of tau/pS422 antibody and reduced progression of tau pathology in a mouse model of Alzheimer’s disease. *Brain* 137 2834–2846. 10.1093/brain/awu213 25085375

[B22] Cooper J. Lathuiliere A. Migliorini M. Arai A. Wani M. Dujardin S. (2020). LRP1 and SORL1 regulate tau internalization and degradation and enhance tau seeding. *bioRxiv* [Preprint]. 10.1101/2020.11.17.386581 PMC8164048 33930462

[B23] Croft C. L. Wade M. A. Kurbatskaya K. Mastrandreas P. Hughes M. M. Phillips E. C. (2017). Membrane association and release of wild-type and pathological tau from organotypic brain slice cultures. *Cell Death Dis.* 8:e2671. 10.1038/cddis.2017.97 28300838 PMC5386587

[B24] Csordas G. Weaver D. Hajnoczky G. (2018). Endoplasmic reticulum-mitochondrial contactology: structure and signaling functions. *Trends Cell Biol.* 28 523–540. 10.1016/j.tcb.2018.02.009 29588129 PMC6005738

[B25] Cutler R. G. Kelly J. Storie K. Pedersen W. A. Tammara A. Hatanpaa K. (2004). Involvement of oxidative stress-induced abnormalities in ceramide and cholesterol metabolism in brain aging and Alzheimer’s disease. *Proc. Natl. Acad. Sci. U. S. A.* 101 2070–2075. 10.1073/pnas.0305799101 14970312 PMC357053

[B26] De Cecco E. Celauro L. Vanni S. Grandolfo M. Bistaffa E. Moda F. (2020). The uptake of tau amyloid fibrils is facilitated by the cellular prion protein and hampers prion propagation in cultured cells. *J. Neurochem.* 155 577–591. 10.1111/jnc.15040 32394432

[B27] De La-Rocque S. Moretto E. Butnaru I. Schiavo G. (2020). Knockin’ on heaven’s door: molecular mechanisms of neuronal tau uptake. *J. Neurochem.* 156 563–588. 10.1111/jnc.15144 32770783 PMC8432157

[B28] Di Paolo G. Kim T. W. (2011). Linking lipids to Alzheimer’s disease: cholesterol and beyond. *Nat. Rev. Neurosci.* 12 284–296. 10.1038/nrn3012 21448224 PMC3321383

[B29] Dieckmann M. Dietrich M. F. Herz J. (2010). Lipoprotein receptors–an evolutionarily ancient multifunctional receptor family. *Biol. Chem.* 391 1341–1363. 10.1515/BC.2010.129 20868222 PMC3529395

[B30] Distl R. Meske V. Ohm T. G. (2001). Tangle-bearing neurons contain more free cholesterol than adjacent tangle-free neurons. *Acta Neuropathol.* 101 547–554. 10.1007/s004010000314 11515782

[B31] Distl R. Treiber-Held S. Albert F. Meske V. Harzer K. Ohm T. G. (2003). Cholesterol storage and tau pathology in Niemann-Pick type C disease in the brain. *J. Pathol.* 200 104–111. 10.1002/path.1320 12692848

[B32] Djelti F. Braudeau J. Hudry E. Dhenain M. Varin J. Bieche I. (2015). CYP46A1 inhibition, brain cholesterol accumulation and neurodegeneration pave the way for Alzheimer’s disease. *Brain* 138 2383–2398. 10.1093/brain/awv166 26141492

[B33] Doktorova M. Heberle F. A. Kingston R. L. Khelashvili G. Cuendet M. A. Wen Y. (2017). Cholesterol promotes protein binding by affecting membrane electrostatics and solvation properties. *Biophys. J.* 113 2004–2015. 10.1016/j.bpj.2017.08.055 29117524 PMC5685651

[B34] Drubin D. G. Kirschner M. W. (1986). Tau protein function in living cells. *J. Cell Biol.* 103 2739–2746. 10.1083/jcb.103.6.2739 3098742 PMC2114585

[B35] Dujardin S. Begard S. Caillierez R. Lachaud C. Delattre L. Carrier S. (2014). Ectosomes: a new mechanism for non-exosomal secretion of tau protein. *PLoS One* 9:e100760. 10.1371/journal.pone.0100760 24971751 PMC4074092

[B36] Eftekharzadeh B. Daigle J. G. Kapinos L. E. Coyne A. Schiantarelli J. Carlomagno Y. (2018). Tau protein disrupts nucleocytoplasmic transport in Alzheimer’s disease. *Neuron* 99:e927. 10.1016/j.neuron.2018.07.039 30189209 PMC6240334

[B37] Eidenmuller J. Fath T. Maas T. Pool M. Sontag E. Brandt R. (2001). Phosphorylation-mimicking glutamate clusters in the proline-rich region are sufficient to simulate the functional deficiencies of hyperphosphorylated tau protein. *Biochem. J.* 357 759–767. 10.1042/0264-6021:3570759 11463346 PMC1222005

[B38] Ekinci F. J. Shea T. B. (2000). Phosphorylation of tau alters its association with the plasma membrane. *Cell. Mol. Neurobiol.* 20 497–508. 10.1023/a:1007075115574 10901269 PMC11537539

[B39] Elbaum-Garfinkle S. Ramlall T. Rhoades E. (2010). The role of the lipid bilayer in tau aggregation. *Biophys. J.* 98 2722–2730. 10.1016/j.bpj.2010.03.013 20513417 PMC2877329

[B40] Esteras N. Kundel F. Amodeo G. F. Pavlov E. V. Klenerman D. Abramov A. Y. (2020). Insoluble tau aggregates induce neuronal death through modification of membrane ion conductance, activation of voltage-gated calcium channels and NADPH oxidase. *FEBS J.* 288 127–141. 10.1111/febs.15340 32338825

[B41] Fanni A. M. Vander Zanden C. M. Majewska P. V. Majewski J. Chi E. Y. (2019). Membrane-mediated fibrillation and toxicity of the tau hexapeptide PHF6. *J. Biol. Chem.* 294 15304–15317. 10.1074/jbc.RA119.010003 31439664 PMC6802503

[B42] Farah C. A. Perreault S. Liazoghli D. Desjardins M. Anton A. Lauzon M. (2006). Tau interacts with Golgi membranes and mediates their association with microtubules. *Cell Motil. Cytoskeleton* 63 710–724. 10.1002/cm.20157 16960886

[B43] Fein J. A. Sokolow S. Miller C. A. Vinters H. V. Yang F. Cole G. M. (2008). Co-localization of amyloid beta and tau pathology in Alzheimer’s disease synaptosomes. *Am. J. Pathol.* 172 1683–1692. 10.2353/ajpath.2008.070829 18467692 PMC2408427

[B44] Friedhoff P. Schneider A. Mandelkow E. M. Mandelkow E. (1998). Rapid assembly of Alzheimer-like paired helical filaments from microtubule-associated protein tau monitored by fluorescence in solution. *Biochemistry* 37 10223–10230. 10.1021/bi980537d 9665729

[B45] Frost B. Jacks R. L. Diamond M. I. (2009). Propagation of tau misfolding from the outside to the inside of a cell. *J. Biol. Chem.* 284 12845–12852. 10.1074/jbc.M808759200 19282288 PMC2676015

[B46] Gauthier-Kemper A. Suarez Alonso M. Sundermann F. Niewidok B. Fernandez M. P. Bakota L. (2018). Annexins A2 and A6 interact with the extreme N terminus of tau and thereby contribute to tau’s axonal localization. *J. Biol. Chem.* 293 8065–8076. 10.1074/jbc.RA117.000490 29636414 PMC5971446

[B47] Gauthier-Kemper A. Weissmann C. Golovyashkina N. Sebo-Lemke Z. Drewes G. Gerke V. (2011). The frontotemporal dementia mutation R406W blocks tau’s interaction with the membrane in an annexin A2-dependent manner. *J. Cell Biol.* 192 647–661. 10.1083/jcb.201007161 21339331 PMC3044115

[B48] Gellermann G. P. Appel T. R. Davies P. Diekmann S. (2006). Paired helical filaments contain small amounts of cholesterol, phosphatidylcholine and sphingolipids. *Biol. Chem.* 387 1267–1274. 10.1515/BC.2006.157 16972796

[B49] Georgieva E. R. Xiao S. Borbat P. P. Freed J. H. Eliezer D. (2014). Tau binds to lipid membrane surfaces via short amphipathic helices located in its microtubule-binding repeats. *Biophys. J.* 107 1441–1452. 10.1016/j.bpj.2014.07.046 25229151 PMC4167292

[B50] Ghribi O. Larsen B. Schrag M. Herman M. M. (2006). High cholesterol content in neurons increases BACE, beta-amyloid, and phosphorylated tau levels in rabbit hippocampus. *Exp. Neurol.* 200 460–467. 10.1016/j.expneurol.2006.03.019 16696972

[B51] Giaccone G. Pedrotti B. Migheli A. Verga L. Perez J. Racagni G. (1996). beta PP and Tau interaction. A possible link between amyloid and neurofibrillary tangles in Alzheimer’s disease. *Am. J. Pathol.* 148 79–87. 8546229 PMC1861592

[B52] Girardot N. Allinquant B. Langui D. Laquerriere A. Dubois B. Hauw J. J. (2003). Accumulation of flotillin-1 in tangle-bearing neurones of Alzheimer’s disease. *Neuropathol. Appl. Neurobiol.* 29 451–461. 10.1046/j.1365-2990.2003.00479.x 14507337

[B53] Glockner F. Meske V. Lutjohann D. Ohm T. G. (2011). Dietary cholesterol and its effect on tau protein: a study in apolipoprotein E-deficient and P301L human tau mice. *J. Neuropathol. Exp. Neurol.* 70 292–301. 10.1097/NEN.0b013e318212f185 21412171

[B54] Glockner F. Ohm T. G. (2014). Tau pathology induces intraneuronal cholesterol accumulation. *J. Neuropathol. Exp. Neurol.* 73 846–854. 10.1097/NEN.0000000000000103 25101701

[B55] Gomez-Ramos A. Diaz-Hernandez M. Cuadros R. Hernandez F. Avila J. (2006). Extracellular tau is toxic to neuronal cells. *FEBS Lett.* 580 4842–4850. 10.1016/j.febslet.2006.07.078 16914144

[B56] Gomez-Ramos A. Diaz-Hernandez M. Rubio A. Miras-Portugal M. T. Avila J. (2008). Extracellular tau promotes intracellular calcium increase through M1 and M3 muscarinic receptors in neuronal cells. *Mol. Cell. Neurosci.* 37 673–681. 10.1016/j.mcn.2007.12.010 18272392

[B57] Gray E. G. Paula-Barbosa M. Roher A. (1987). Alzheimer’s disease: paired helical filaments and cytomembranes. *Neuropathol. Appl. Neurobiol.* 13 91–110. 10.1111/j.1365-2990.1987.tb00174.x 3614544

[B58] Guix F. X. Corbett G. T. Cha D. J. Mustapic M. Liu W. Mengel D. (2018). Detection of aggregation-competent tau in neuron-derived extracellular vesicles. *Int. J. Mol. Sci.* 19:663. 10.3390/ijms19030663 29495441 PMC5877524

[B59] Han J. Zhang J. Yao H. Wang X. Li F. Chen L. (2006). Study on interaction between microtubule associated protein tau and prion protein. *Sci. China C Life Sci.* 49 473–479. 10.1007/s11427-006-2019-9 17172055

[B60] Henkins K. M. Sokolow S. Miller C. A. Vinters H. V. Poon W. W. Cornwell L. B. (2012). Extensive p-tau pathology and SDS-stable p-tau oligomers in Alzheimer’s cortical synapses. *Brain Pathol.* 22 826–833. 10.1111/j.1750-3639.2012.00598.x 22486774 PMC3410970

[B61] Herz J. Hamann U. Rogne S. Myklebost O. Gausepohl H. Stanley K. K. (1988). Surface location and high affinity for calcium of a 500-kd liver membrane protein closely related to the LDL-receptor suggest a physiological role as lipoprotein receptor. *EMBO J.* 7 4119–4127. 3266596 10.1002/j.1460-2075.1988.tb03306.x PMC455121

[B62] Holmes B. B. Devos S. L. Kfoury N. Li M. Jacks R. Yanamandra K. (2013). Heparan sulfate proteoglycans mediate internalization and propagation of specific proteopathic seeds. *Proc. Natl. Acad. Sci. U. S. A.* 110 E3138–E3147. 10.1073/pnas.1301440110 23898162 PMC3746848

[B63] Hudak A. Kusz E. Domonkos I. Josvay K. Kodamullil A. T. Szilak L. (2019). Contribution of syndecans to cellular uptake and fibrillation of alpha-synuclein and tau. *Sci. Rep.* 9:16543. 10.1038/s41598-019-53038-z 31719623 PMC6851098

[B64] Iozzo R. V. (1998). Matrix proteoglycans: from molecular design to cellular function. *Annu. Rev. Biochem.* 67 609–652. 10.1146/annurev.biochem.67.1.609 9759499

[B65] Islam K. Levy E. (1997). Carboxyl-terminal fragments of beta-amyloid precursor protein bind to microtubules and the associated protein tau. *Am. J. Pathol.* 151 265–271. 9212751 PMC1857905

[B66] Jancsik V. Filliol D. Felter S. Rendon A. (1989). Binding of microtubule-associated proteins (MAPs) to rat brain mitochondria: a comparative study of the binding of MAP2, its microtubule-binding and projection domains, and tau proteins. *Cell Motil. Cytoskeleton* 14 372–381. 10.1002/cm.970140307 2510942

[B67] Jeganathan S. Von Bergen M. Mandelkow E. M. Mandelkow E. (2008). The natively unfolded character of tau and its aggregation to Alzheimer-like paired helical filaments. *Biochemistry* 47 10526–10539. 10.1021/bi800783d 18783251

[B68] Jones E. M. Dubey M. Camp P. J. Vernon B. C. Biernat J. Mandelkow E. (2012). Interaction of tau protein with model lipid membranes induces tau structural compaction and membrane disruption. *Biochemistry* 51 2539–2550. 10.1021/bi201857v 22401494 PMC3319454

[B69] Kampers T. Friedhoff P. Biernat J. Mandelkow E. M. Mandelkow E. (1996). RNA stimulates aggregation of microtubule-associated protein tau into Alzheimer-like paired helical filaments. *FEBS Lett.* 399 344–349. 10.1016/s0014-5793(96)01386-5 8985176

[B70] Kanekiyo T. Bu G. (2014). The low-density lipoprotein receptor-related protein 1 and amyloid-beta clearance in Alzheimer’s disease. *Front. Aging Neurosci.* 6:93. 10.3389/fnagi.2014.00093 24904407 PMC4033011

[B71] Karch C. M. Jeng A. T. Goate A. M. (2012). Extracellular Tau levels are influenced by variability in Tau that is associated with tauopathies. *J. Biol. Chem.* 287 42751–42762. 10.1074/jbc.M112.380642 23105105 PMC3522274

[B72] Katsinelos T. Zeitler M. Dimou E. Karakatsani A. Muller H. M. Nachman E. (2018). Unconventional secretion mediates the trans-cellular spreading of tau. *Cell Rep.* 23 2039–2055. 10.1016/j.celrep.2018.04.056 29768203

[B73] Kawarabayashi T. Shoji M. Younkin L. H. Wen-Lang L. Dickson D. W. Murakami T. (2004). Dimeric amyloid beta protein rapidly accumulates in lipid rafts followed by apolipoprotein E and phosphorylated tau accumulation in the Tg2576 mouse model of Alzheimer’s disease. *J. Neurosci.* 24 3801–3809. 10.1523/JNEUROSCI.5543-03.2004 15084661 PMC6729359

[B74] Kim J. Basak J. M. Holtzman D. M. (2009). The role of apolipoprotein E in Alzheimer’s disease. *Neuron* 63 287–303. 10.1016/j.neuron.2009.06.026 19679070 PMC3044446

[B75] King M. E. Gamblin T. C. Kuret J. Binder L. I. (2000). Differential assembly of human tau isoforms in the presence of arachidonic acid. *J. Neurochem.* 74 1749–1757. 10.1046/j.1471-4159.2000.0741749.x 10737634

[B76] Klein C. Kramer E. M. Cardine A. M. Schraven B. Brandt R. Trotter J. (2002). Process outgrowth of oligodendrocytes is promoted by interaction of fyn kinase with the cytoskeletal protein tau. *J. Neurosci.* 22 698–707. 10.1523/JNEUROSCI.22-03-00698.2002 11826099 PMC6758498

[B77] Kosik K. S. Joachim C. L. Selkoe D. J. (1986). Microtubule-associated protein tau (tau) is a major antigenic component of paired helical filaments in Alzheimer disease. *Proc. Natl. Acad. Sci. U. S. A.* 83 4044–4048. 10.1073/pnas.83.11.4044 2424016 PMC323662

[B78] Kunze G. Barre P. Scheidt H. A. Thomas L. Eliezer D. Huster D. (2012). Binding of the three-repeat domain of tau to phospholipid membranes induces an aggregated-like state of the protein. *Biochim. Biophys. Acta* 1818 2302–2313. 10.1016/j.bbamem.2012.03.019 22521809 PMC3595127

[B79] Lazar A. N. Bich C. Panchal M. Desbenoit N. Petit V. W. Touboul D. (2013). Time-of-flight secondary ion mass spectrometry (TOF-SIMS) imaging reveals cholesterol overload in the cerebral cortex of Alzheimer disease patients. *Acta Neuropathol.* 125 133–144. 10.1007/s00401-012-1041-1 22956244

[B80] Lee G. Newman S. T. Gard D. L. Band H. Panchamoorthy G. (1998). Tau interacts with src-family non-receptor tyrosine kinases. *J. Cell Sci.* 111 (Pt 21) 3167–3177. 9763511 10.1242/jcs.111.21.3167

[B81] Lee S. Kim W. Li Z. Hall G. F. (2012). Accumulation of vesicle-associated human tau in distal dendrites drives degeneration and tau secretion in an in situ cellular tauopathy model. *Int. J. Alzheimers Dis.* 2012 172837. 10.1155/2012/172837 22315694 PMC3270555

[B82] Lindwall G. Cole R. D. (1984). Phosphorylation affects the ability of tau protein to promote microtubule assembly. *J. Biol. Chem.* 259 5301–5305. 6425287

[B83] Lira-De León K. I. De Anda-Hernández M. A. Campos-Peña V. Meraz-Ríos M. A. (2009). “Plasma membrane-associated PHF-core could be the trigger for tau aggregation in alzheimer’s disease,” in *Current Hypotheses and Research Milestones in Alzheimer’s Disease*, eds Perry G. P. Maccioni R. B. (Boston, MA: Springer), 93–100.

[B84] LoPresti P. Szuchet S. Papasozomenos S. C. Zinkowski R. P. Binder L. I. (1995). Functional implications for the microtubule-associated protein tau: localization in oligodendrocytes. *Proc. Natl. Acad. Sci. U. S. A.* 92 10369–10373. 10.1073/pnas.92.22.10369 7479786 PMC40798

[B85] Maas T. Eidenmuller J. Brandt R. (2000). Interaction of tau with the neural membrane cortex is regulated by phosphorylation at sites that are modified in paired helical filaments. *J. Biol. Chem.* 275 15733–15740. 10.1074/jbc.M000389200 10747907

[B86] Majewski J. Jones E. M. Vander Zanden C. M. Biernat J. Mandelkow E. Chi E. Y. (2020). Lipid membrane templated misfolding and self-assembly of intrinsically disordered tau protein. *Sci. Rep.* 10:13324. 10.1038/s41598-020-70208-6 32770092 PMC7414892

[B87] Mari S. A. Wegmann S. Tepper K. Hyman B. T. Mandelkow E. M. Mandelkow E. (2018). Reversible cation-selective attachment and self-assembly of human tau on supported brain lipid membranes. *Nano Lett.* 18 3271–3281. 10.1021/acs.nanolett.8b01085 29644863 PMC6588182

[B88] Marquer C. Laine J. Dauphinot L. Hanbouch L. Lemercier-Neuillet C. Pierrot N. (2014). Increasing membrane cholesterol of neurons in culture recapitulates Alzheimer’s disease early phenotypes. *Mol. Neurodegener.* 9:60. 10.1186/1750-1326-9-60 25524049 PMC4280040

[B89] Martin L. Latypova X. Terro F. (2011). Post-translational modifications of tau protein: implications for Alzheimer’s disease. *Neurochem. Int.* 58 458–471. 10.1016/j.neuint.2010.12.023 21215781

[B90] Matsumoto S. E. Motoi Y. Ishiguro K. Tabira T. Kametani F. Hasegawa M. (2015). The twenty-four KDa C-terminal tau fragment increases with aging in tauopathy mice: implications of prion-like properties. *Hum. Mol. Genet.* 24 6403–6416. 10.1093/hmg/ddv351 26374846

[B91] Merezhko M. Brunello C. A. Yan X. Vihinen H. Jokitalo E. Uronen R. L. (2018). Secretion of tau via an unconventional non-vesicular mechanism. *Cell Rep.* 25:e2024. 10.1016/j.celrep.2018.10.078 30463001

[B92] Mirbaha H. Holmes B. B. Sanders D. W. Bieschke J. Diamond M. I. (2015). Tau trimers are the minimal propagation unit spontaneously internalized to seed intracellular aggregation. *J. Biol. Chem.* 290 14893–14903. 10.1074/jbc.M115.652693 25887395 PMC4463437

[B93] Moltedo O. Remondelli P. Amodio G. (2019). The mitochondria-endoplasmic reticulum contacts and their critical role in aging and age-associated diseases. *Front. Cell Dev. Biol.* 7:172. 10.3389/fcell.2019.00172 31497601 PMC6712070

[B94] Morozova V. Cohen L. S. Makki A. E. Shur A. Pilar G. El Idrissi A. (2019). Normal and pathological tau uptake mediated by M1/M3 muscarinic receptors promotes opposite neuronal changes. *Front. Cell. Neurosci.* 13:403. 10.3389/fncel.2019.00403 31555098 PMC6737038

[B95] Morris M. Maeda S. Vossel K. Mucke L. (2011). The many faces of tau. *Neuron* 70 410–426. 10.1016/j.neuron.2011.04.009 21555069 PMC3319390

[B96] Mukrasch M. D. Bibow S. Korukottu J. Jeganathan S. Biernat J. Griesinger C. (2009). Structural polymorphism of 441-residue tau at single residue resolution. *PLoS Biol.* 7:e34. 10.1371/journal.pbio.1000034 19226187 PMC2642882

[B97] Nicholson A. M. Ferreira A. (2009). Increased membrane cholesterol might render mature hippocampal neurons more susceptible to beta-amyloid-induced calpain activation and tau toxicity. *J. Neurosci.* 29 4640–4651. 10.1523/JNEUROSCI.0862-09.2009 19357288 PMC2705291

[B98] Nicholson A. M. Methner D. N. Ferreira A. (2011). Membrane cholesterol modulates {beta}-amyloid-dependent tau cleavage by inducing changes in the membrane content and localization of N-methyl-D-aspartic acid receptors. *J. Biol. Chem.* 286 976–986. 10.1074/jbc.M110.154138 21047784 PMC3020782

[B99] Nuebling G. S. Plesch E. Ruf V. C. Hogen T. Lorenzl S. Kamp F. (2020). Binding of metal-ion-induced tau oligomers to lipid surfaces is enhanced by GSK-3beta-mediated phosphorylation. *ACS Chem. Neurosci.* 11 880–887. 10.1021/acschemneuro.9b00459 32069020

[B100] Pawelec P. Ziemka-Nalecz M. Sypecka J. Zalewska T. (2020). The impact of the CX3CL1/CX3CR1 axis in neurological disorders. *Cells* 9:2277. 10.3390/cells9102277 33065974 PMC7600611

[B101] Perea J. R. Lopez E. Diez-Ballesteros J. C. Avila J. Hernandez F. Bolos M. (2019). Extracellular monomeric tau is internalized by astrocytes. *Front. Neurosci.* 13:442. 10.3389/fnins.2019.00442 31118883 PMC6504834

[B102] Pernegre C. Duquette A. Leclerc N. (2019). Tau secretion: good and bad for neurons. *Front. Neurosci.* 13:649. 10.3389/fnins.2019.00649 31293374 PMC6606725

[B103] Perreault S. Bousquet O. Lauzon M. Paiement J. Leclerc N. (2009). Increased association between rough endoplasmic reticulum membranes and mitochondria in transgenic mice that express P301L tau. *J. Neuropathol. Exp. Neurol.* 68 503–514. 10.1097/NEN.0b013e3181a1fc49 19525898

[B104] Piacentini R. Li Puma D. D. Mainardi M. Lazzarino G. Tavazzi B. Arancio O. (2017). Reduced gliotransmitter release from astrocytes mediates tau-induced synaptic dysfunction in cultured hippocampal neurons. *Glia* 65 1302–1316. 10.1002/glia.23163 28519902 PMC5520670

[B105] Plouffe V. Mohamed N. V. Rivest-Mcgraw J. Bertrand J. Lauzon M. Leclerc N. (2012). Hyperphosphorylation and cleavage at D421 enhance tau secretion. *PLoS One* 7:e36873. 10.1371/journal.pone.0036873 22615831 PMC3352936

[B106] Pooler A. M. Phillips E. C. Lau D. H. Noble W. Hanger D. P. (2013). Physiological release of endogenous tau is stimulated by neuronal activity. *EMBO Rep.* 14 389–394. 10.1038/embor.2013.15 23412472 PMC3615658

[B107] Pooler A. M. Usardi A. Evans C. J. Philpott K. L. Noble W. Hanger D. P. (2012). Dynamic association of tau with neuronal membranes is regulated by phosphorylation. *Neurobiol. Aging* 33 e427–e438. 10.1016/j.neurobiolaging.2011.01.005 21388709

[B108] Puangmalai N. Bhatt N. Montalbano M. Sengupta U. Gaikwad S. Ventura F. (2020). Internalization mechanisms of brain-derived tau oligomers from patients with Alzheimer’s disease, progressive supranuclear palsy and dementia with Lewy bodies. *Cell Death Dis.* 11:314. 10.1038/s41419-020-2503-3 32366836 PMC7198578

[B109] Puzzo D. Piacentini R. Fa M. Gulisano W. Li Puma D. D. Staniszewski A. (2017). LTP and memory impairment caused by extracellular Abeta and Tau oligomers is APP-dependent. *Elife* 6:e26991. 10.7554/eLife.26991 28696204 PMC5529106

[B110] Rahman A. Akterin S. Flores-Morales A. Crisby M. Kivipelto M. Schultzberg M. (2005). High cholesterol diet induces tau hyperphosphorylation in apolipoprotein E deficient mice. *FEBS Lett.* 579 6411–6416. 10.1016/j.febslet.2005.10.024 16288750

[B111] Rauch J. N. Chen J. J. Sorum A. W. Miller G. M. Sharf T. See S. K. (2018). Tau internalization is regulated by 6-O Sulfation on Heparan Sulfate Proteoglycans (HSPGs). *Sci. Rep.* 8:6382. 10.1038/s41598-018-24904-z 29686391 PMC5913225

[B112] Rauch J. N. Luna G. Guzman E. Audouard M. Challis C. Sibih Y. E. (2020). LRP1 is a master regulator of tau uptake and spread. *Nature* 580 381–385. 10.1038/s41586-020-2156-5 32296178 PMC7687380

[B113] Rendon A. Jung D. Jancsik V. (1990). Interaction of microtubules and microtubule-associated proteins (MAPs) with rat brain mitochondria. *Biochem. J.* 269 555–556. 10.1042/bj2690555 2386493 PMC1131616

[B114] Sarrazin S. Lamanna W. C. Esko J. D. (2011). Heparan sulfate proteoglycans. *Cold Spring Harb. Perspect. Biol.* 3:a004952. 10.1101/cshperspect.a004952 21690215 PMC3119907

[B115] Seidler P. M. Boyer D. R. Rodriguez J. A. Sawaya M. R. Cascio D. Murray K. (2018). Structure-based inhibitors of tau aggregation. *Nat. Chem.* 10 170–176. 10.1038/nchem.2889 29359764 PMC5784779

[B116] Sepulveda-Diaz J. E. Alavi Naini S. M. Huynh M. B. Ouidja M. O. Yanicostas C. Chantepie S. (2015). HS3ST2 expression is critical for the abnormal phosphorylation of tau in Alzheimer’s disease-related tau pathology. *Brain* 138 1339–1354. 10.1093/brain/awv056 25842390 PMC5963411

[B117] Shelton S. B. Johnson G. V. (2001). Tau and HMW tau phosphorylation and compartmentalization in apoptotic neuronal PC12 cells. *J. Neurosci. Res.* 66 203–213. 10.1002/jnr.1212 11592115

[B118] Shobab L. A. Hsiung G. Y. Feldman H. H. (2005). Cholesterol in Alzheimer’s disease. *Lancet. Neurol.* 4 841–852. 10.1016/S1474-4422(05)70248-9 16297842

[B119] Shrivastava A. N. Redeker V. Pieri L. Bousset L. Renner M. Madiona K. (2019). Clustering of Tau fibrils impairs the synaptic composition of alpha3-Na(+)/K(+)-ATPase and AMPA receptors. *EMBO J.* 38:e99871. 10.15252/embj.201899871 30630857 PMC6356061

[B120] Simic G. Babic Leko M. Wray S. Harrington C. Delalle I. Jovanov-Milosevic N. (2016). Tau protein hyperphosphorylation and aggregation in Alzheimer’s disease and other tauopathies, and possible neuroprotective strategies. *Biomolecules* 6:6. 10.3390/biom6010006 26751493 PMC4808800

[B121] Simon D. Garcia-Garcia E. Royo F. Falcon-Perez J. M. Avila J. (2012). Proteostasis of tau. Tau overexpression results in its secretion via membrane vesicles. *FEBS Lett.* 586 47–54. 10.1016/j.febslet.2011.11.022 22138183

[B122] Smith M. A. Siedlak S. L. Richey P. L. Mulvihill P. Ghiso J. Frangione B. (1995). Tau protein directly interacts with the amyloid beta-protein precursor: implications for Alzheimer’s disease. *Nat. Med.* 1 365–369. 10.1038/nm0495-365 7585068

[B123] Spoelgen R. Adams K. W. Koker M. Thomas A. V. Andersen O. M. Hallett P. J. (2009). Interaction of the apolipoprotein E receptors low density lipoprotein receptor-related protein and sorLA/LR11. *Neuroscience* 158 1460–1468. 10.1016/j.neuroscience.2008.10.061 19047013 PMC2709796

[B124] Stopschinski B. E. Holmes B. B. Miller G. M. Manon V. A. Vaquer-Alicea J. Prueitt W. L. (2018). Specific glycosaminoglycan chain length and sulfation patterns are required for cell uptake of tau versus alpha-synuclein and beta-amyloid aggregates. *J. Biol. Chem.* 293 10826–10840. 10.1074/jbc.RA117.000378 29752409 PMC6036193

[B125] Szabo L. Eckert A. Grimm A. (2020). Insights into disease-associated tau impact on mitochondria. *Int. J. Mol. Sci.* 21:6344. 10.3390/ijms21176344 32882957 PMC7503371

[B126] Tai H. C. Serrano-Pozo A. Hashimoto T. Frosch M. P. Spires-Jones T. L. Hyman B. T. (2012). The synaptic accumulation of hyperphosphorylated tau oligomers in Alzheimer disease is associated with dysfunction of the ubiquitin-proteasome system. *Am. J. Pathol.* 181 1426–1435. 10.1016/j.ajpath.2012.06.033 22867711 PMC3463637

[B127] Tajima Y. Ishikawa M. Maekawa K. Murayama M. Senoo Y. Nishimaki-Mogami T. (2013). Lipidomic analysis of brain tissues and plasma in a mouse model expressing mutated human amyloid precursor protein/tau for Alzheimer’s disease. *Lipids Health Dis.* 12:68. 10.1186/1476-511X-12-68 23659495 PMC3668217

[B128] Takahashi M. Miyata H. Kametani F. Nonaka T. Akiyama H. Hisanaga S. (2015). Extracellular association of APP and tau fibrils induces intracellular aggregate formation of tau. *Acta Neuropathol.* 129 895–907. 10.1007/s00401-015-1415-2 25869641 PMC4436700

[B129] Usardi A. Pooler A. M. Seereeram A. Reynolds C. H. Derkinderen P. Anderton B. (2011). Tyrosine phosphorylation of tau regulates its interactions with Fyn SH2 domains, but not SH3 domains, altering the cellular localization of tau. *FEBS J.* 278 2927–2937. 10.1111/j.1742-4658.2011.08218.x 21692989

[B130] van der Kant R. Langness V. F. Herrera C. M. Williams D. A. Fong L. K. Leestemaker Y. (2019). Cholesterol metabolism is a druggable axis that independently regulates tau and amyloid-beta in iPSC-derived Alzheimer’s disease neurons. *Cell Stem Cell* 24:e369. 10.1016/j.stem.2018.12.013 30686764 PMC6414424

[B131] Vogels T. Leuzy A. Cicognola C. Ashton N. J. Smolek T. Novak M. (2020). Propagation of tau pathology: integrating insights from postmortem and in vivo studies. *Biol. Psychiatry* 87 808–818. 10.1016/j.biopsych.2019.09.019 31735253

[B132] von Bergen M. Barghorn S. Li L. Marx A. Biernat J. Mandelkow E. M. (2001). Mutations of tau protein in frontotemporal dementia promote aggregation of paired helical filaments by enhancing local beta-structure. *J. Biol. Chem.* 276 48165–48174. 10.1074/jbc.M105196200 11606569

[B133] von Bergen M. Friedhoff P. Biernat J. Heberle J. Mandelkow E. M. Mandelkow E. (2000). Assembly of tau protein into Alzheimer paired helical filaments depends on a local sequence motif ((306)VQIVYK(311)) forming beta structure. *Proc. Natl. Acad. Sci. U. S. A.* 97 5129–5134. 10.1073/pnas.97.10.5129 10805776 PMC25793

[B134] Walter J. van Echten-Deckert G. (2013). Cross-talk of membrane lipids and Alzheimer-related proteins. *Mol. Neurodegener.* 8:34. 10.1186/1750-1326-8-34 24148205 PMC4016522

[B135] Wang P. Ding K. (2014). Proteoglycans and glycosaminoglycans in misfolded proteins formation in Alzheimer’s disease. *Protein Pept. Lett.* 21 1048–1056. 10.2174/0929866521666140626095145 24975673

[B136] Wang W. Zhao F. Ma X. Perry G. Zhu X. (2020). Mitochondria dysfunction in the pathogenesis of Alzheimer’s disease: recent advances. *Mol. Neurodegener.* 15:30. 10.1186/s13024-020-00376-6 32471464 PMC7257174

[B137] Wang X. F. Dong C. F. Zhang J. Wan Y. Z. Li F. Huang Y. X. (2008). Human tau protein forms complex with PrP and some GSS- and fCJD-related PrP mutants possess stronger binding activities with tau in vitro. *Mol. Cell. Biochem.* 310 49–55. 10.1007/s11010-007-9664-6 18038270

[B138] Wang Y. Balaji V. Kaniyappan S. Kruger L. Irsen S. Tepper K. (2017). The release and trans-synaptic transmission of Tau via exosomes. *Mol. Neurodegener.* 12:5. 10.1186/s13024-016-0143-y 28086931 PMC5237256

[B139] Wang Y. Mandelkow E. (2016). Tau in physiology and pathology. *Nat. Rev. Neurosci.* 17 5–21. 10.1038/nrn.2015.1 26631930

[B140] Wang Y. Martinez-Vicente M. Kruger U. Kaushik S. Wong E. Mandelkow E. M. (2009). Tau fragmentation, aggregation and clearance: the dual role of lysosomal processing. *Hum. Mol. Genet.* 18 4153–4170. 10.1093/hmg/ddp367 19654187 PMC2758146

[B141] Wegmann S. Nicholls S. Takeda S. Fan Z. Hyman B. T. (2016). Formation, release, and internalization of stable tau oligomers in cells. *J. Neurochem.* 139 1163–1174. 10.1111/jnc.13866 27731899 PMC5283951

[B142] Weingarten M. D. Lockwood A. H. Hwo S. Y. Kirschner M. W. (1975). A protein factor essential for microtubule assembly. *Proc. Natl. Acad. Sci. U. S. A.* 72 1858–1862. 10.1073/pnas.72.5.1858 1057175 PMC432646

[B143] Wray S. Noble W. (2009). Linking amyloid and tau pathology in Alzheimer’s disease: the role of membrane cholesterol in Abeta-mediated tau toxicity. *J. Neurosci.* 29 9665–9667. 10.1523/JNEUROSCI.2234-09.2009 19657019 PMC6666583

[B144] Xu D. Esko J. D. (2014). Demystifying heparan sulfate-protein interactions. *Annu. Rev. Biochem.* 83 129–157. 10.1146/annurev-biochem-060713-035314 24606135 PMC7851832

[B145] Yan X. Nykanen N. P. Brunello C. A. Haapasalo A. Hiltunen M. Uronen R. L. (2016). FRMD4A-cytohesin signaling modulates the cellular release of tau. *J. Cell Sci.* 129 2003–2015. 10.1242/jcs.180745 27044754

[B146] Zhang Y. Zhao Y. Zhang L. Yu W. Wang Y. Chang W. (2019). Cellular prion protein as a receptor of toxic amyloid-beta42 oligomers is important for Alzheimer’s disease. *Front. Cell. Neurosci.* 13:339. 10.3389/fncel.2019.00339 31417361 PMC6682659

[B147] Zhao J. Zhu Y. Song X. Xiao Y. Su G. Liu X. (2020). 3-O-sulfation of heparan sulfate enhances tau interaction and cellular uptake. *Angew. Chem. Int. Ed. Engl.* 59 1818–1827. 10.1002/anie.201913029 31692167 PMC6982596

[B148] Zhou L. Mcinnes J. Wierda K. Holt M. Herrmann A. G. Jackson R. J. (2017). Tau association with synaptic vesicles causes presynaptic dysfunction. *Nat. Commun.* 8:15295. 10.1038/ncomms15295 28492240 PMC5437271

[B149] Zollo A. Allen Z. Rasmussen H. F. Iannuzzi F. Shi Y. Larsen A. (2017). Sortilin-related receptor expression in human neural stem cells derived from Alzheimer’s disease patients carrying the APOE epsilon 4 allele. *Neural. Plast.* 2017:1892612. 10.1155/2017/1892612 28634550 PMC5467336

